# Development of Immature CD4^+^CD8^+^T Cells Into Mature CD4^+^ T Cells Requires Alpha-1 Antitrypsin as Observed by Treatment in HIV-1 Infected and Uninfected Controls

**DOI:** 10.3389/fcell.2019.00278

**Published:** 2019-11-21

**Authors:** Cynthia L. Bristow, Sara Ferrando-Martinez, Ezequiel Ruiz-Mateos, Manuel Leal, Ronald Winston

**Affiliations:** ^1^Alpha-1 Biologics, Long Island High Technology Incubator, Stony Brook University, Stony Brook, NY, United States; ^2^Institute for Human Genetics and Biochemistry, Geneva, Switzerland; ^3^Immunology Laboratory, Vaccine Research Center (VRC/National Institute of Allergy and Infectious Diseases/National Institutes of Health), Bethesda, MD, United States; ^4^MedImmune, Gaithersburg, MD, United States; ^5^Laboratory of Immunovirology, Clinic Unit of Infectious Diseases, Microbiology and Preventive Medicine, Institute of Biomedicine of Seville, IBiS, University Hospital/CSIC/University of Seville, Seville, Spain

**Keywords:** HIV vaccine, CD4, leukocyte elastase, LDL, cellular locomotion, T cell, thymopoiesis, antitrypsin

## Abstract

Immune cells are, by default, migratory cells that traverse tissue for the purpose of carrying out recognition and recruitment in pathologic inflammation and infection. Members of the LDL receptor family (LDL-RFMs) interact with human leukocyte elastase on the cell surface (HLE-CS) in complex with the abundant blood protein α1proteinase inhibitor (α1PI, α1-antitrypsin, Alpha-1), a process that induces internalization of aggregated functionally-related receptors, including CD4 and the T cell antigen receptor, while simultaneously promoting cellular locomotion. We sought to determine whether augmenting α1PI blood concentration would promote the locomotion of immature T cells through the thymus and generate new CD4^+^ T cells. Two small clinical trials (NCT01370018, NCT01731691, https://clinicaltrials.gov) were conducted in which HIV-1 infected and uninfected individuals were augmented with α1PI and compared with placebo-treated subjects and untreated controls. Blood cell phenotypes were monitored weekly. We found that CD4/CD8 ratio was significantly increased by α1PI augmentation in both uninfected and HIV-1 infected individuals. We found that maturation of CD4^+^CD8^+^ T cells to become immunologically competent CD4^+^ T cells was regulated by α1PI. We propose a strategy targeting HLE-CS for treating secondary immunodeficiency for which there is currently no direct treatment. Treatment to directly elevate T cells in patients with secondary immunodeficiency, including HIV disease, can be provided by alpha-1 antitrypsin augmentation or small molecules that target HLE-CS. Because individuals infected with HIV-1 produce a monoclonal antibody, 3F5, which binds to and inactivates α1PI, a process that prevents α1PI from binding to HLE-CS, thereby blocking locomotion of immature T cells through the thymus to generate CD4^+^ T cells, we further propose that HIV-1 vaccination should include induction of an antibody that binds to and blocks 3F5 activity, thereby preventing AIDS in addition to the current vaccine strategy for preventing HIV-1 infection.

## Introduction

There are many receptor-proteinase inhibitor pairs that induce cellular locomotion, one of which involves the protein encoded by SERPINA1 (α1proteinase inhibitor, α1PI, α1antitrypsin, AAT, Alpha-1) which is normally found at much greater concentration than any other proteinase inhibitor. A primary physiological role for α1PI is to induce cellular locomotion through tissue by binding to human leukocyte elastase on the cell surface (HLE-CS). When LDL receptor family members (LDL-RFMs) on the cell surface form a complex with α1PI and HLE-CS, endocytosis is induced, which results in internalization of adhesion molecules at the trailing edge of a migrating cell thereby propelling a cell forward as it traverses tissue (Schmitz et al., 2003; Bristow et al., 2013). In physiological conditions, subsequent to endocytosis, recycling receptors that are aggregated with LDL-RFMs are returned to the cell surface in early endosomes for reuse. In pathological conditions, α1PI is inactivated by proteinases other than elastase and immune cell locomotion halts precisely at the site of infection or inflammation. This mechanism includes the locomotion of progenitor cells through thymic tissue and the generation of new CD4^+^ T cells in mice and humans (Spurll and Owen, 1981; Owen and Riblet, 1984; Bristow and Flood, 1993). In the absence of sufficient functional α1PI, immature T cells poorly migrate through the thymus to generate new CD4^+^ T cells.

**Figure 1:**
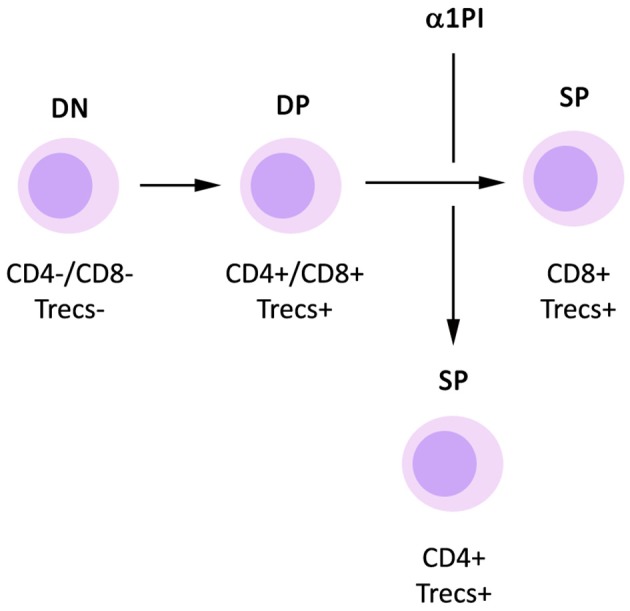
Influence of α1PI on thymopoiesis. By default, DP T cells mature to become CD8^+^ SP T cells. Signaling induced by binding of α1PI to HLE-CS on DP T cells induces NFκB phosphorylation and stimulates the maturation of DP T cells to become CD4^+^ SP T cells.

Generation of new CD4^+^ T helper cells from progenitor cells occurs in known discrete steps (Figure 1). When CD34^+^ hematopoietic progenitor cells are released from bone marrow and during the process of traversing tissue, they mature to become CD25^+^ pre-T cells which do not express CD4 or CD8 on the cell surface, accordingly termed double negatives (DNs). These DNs also do not express the T cell antigen receptor (TcR) or its coreceptor CD3 on the cell surface. After arriving in the thymus, DNs mature through four distinct stages to express both CD4 and CD8 on the cell surface, termed double positives (DPs). In the process of maturing from DNs to DPs, the TcR β-chain rearranges forming cytoplasmic DNA fragments termed β-chain T cell excision circles (β-TRECs). Subsequently, the TcR α-chain rearranges to form signal joint TRECs (sj-TRECs). Once these TcR chains are assembled intracellularly, the intact TcR is expressed on the cell surface in tandem with CD3. Due to this process, the sj/β-TREC ratio has been shown to be useful for quantitating the maturation of DNs to DPs (Ferrando-Martínez et al., 2010). DPs mature to become single positive T cells (SPs) that express either CD4 or CD8 on the cell surface. These SPs undergo positive and negative selection within the thymus via antigen recognition avidity and are released into blood and tissue where they generally act as effector helper T cells or killer T cells, respectively. Total thymic output is relatively invariable in an individual, and by default, DPs mature to become CD8^+^ T cells. However, when DPs do not mature to CD8^+^ T cells, they become CD4^+^ T cells (McDonagh and Bell, 1995).

Clinically, α1PI deficiency (genetic or acquired) is an underlying condition in chronic obstructive pulmonary disease (COPD), a common symptom of HIV-1 disease consistent with disease attendant acquired α1PI deficiency (Bristow et al., 2001). Interestingly, patients with genetic α1PI deficiency have relatively normal numbers of CD4^+^ T cells (Bristow et al., 2012). This is thought to be due to the normal production and secretion of α1PI by cells within lymphoid tissues where α1PI protein synthesis is initiated using different promoters in contrast to those used by liver cells where secretion is severely inhibited (Gullberg et al., 1997; OMIM, 2019). This concept is supported by the presence of detectable α1PI (2–5 μM) in genetically deficient patients, representing 10% of the normal reference range (18–53 μM) (Bristow et al., 2001). An alternative explanation is that similar receptor-proteinase inhibitor pairs (or receptor-proteinase pairs) could substitute for the funtions of the α1PI-HLE pairs.

While COPD often manifests in HIV-1 disease, the principal pathology is loss of CD4^+^ T cells, and this has been attributed to both increased CD4^+^ T cell death and decreased generation of new CD4^+^ T cells (Ribeiro et al., 2002; Dion et al., 2006; Bristow et al., 2010, 2013). The failure of CD4^+^ T cell renewal in HIV-1 infected individuals has been linked to acquired α1PI deficiency which is caused by one monoclonal antibody, 3F5, with specificity for HIV-1 envelope protein gp120 (Bristow et al., 2010, 2012).

Current approaches to HIV-1 vaccine development are to induce broadly neutralizing antibodies (bNAbs) to block direct fusion of the virus to a cell's plasma membrane. However, microscopic imaging has indisputably demonstrated HIV-1 fusing with the plasma membrane within endosomal vesicles, a process that sequesters virions from bNAbs (Hubner et al., 2009; Miyauchi et al., 2009; Bristow et al., 2013; Herold et al., 2014; Law et al., 2016). CD4-coupled HIV-1 has been demonstrated to be internalized via endocytosis mediated by LDL-RFMs (Bristow et al., 2003, 2013).

Many bNABs (e.g., 2F5 and 4E10) additionally bind to human proteins and may also induce autoimmunity. The possibility of such autoimmunity has been largely dismissed as too daunting to consider and an improbable outcome in HIV-1 vaccine design (Haynes et al., 2005). However, 3F5 (same epitope recognition as 1C1), has specificity for HIV-1 envelope protein gp120 and produces autoimmunity in humans (Los Alamos National Laboratory, 2009; Bristow et al., 2012). In the absence of viral particles, 3F5 binds to and inactivates human α1PI, an abundant blood protein (Uhlen et al., 2010; Uhlén et al., 2015; Bristow et al., 2012). In the early stages of HIV-1 disease, 90% of HIV-1 patients have detectable 3F5-induced α1PI deficiency (Bristow et al., 2001). Thus, α1PI is an inadvertant autoimmune target of the HIV-1 antibody response.

We proposed that 3F5-mediated inactivation of α1PI impedes endocytosis thereby thwarting the movement of cells through the thymus and blocking generation of CD4^+^ T cells (Bristow et al., 2012). This hypothesis suggests that α1PI augmentation therapy might increase the number of CD4^+^ T cells in blood. We demonstrate herein that α1PI augmentation increases the number of CD4^+^ T cells in blood by increasing the percentage of DPs that mature to become CD4^+^ T cells during thymopoiesis via a mechansim involving CD3^+^HLE^+^ thymocytes.

## Materials and Methods

### Clinical Trial NCT01370018

This clinical trial had one treatment arm. Using the empirical correlation between α1PI and CD4^+^ lymphocytes, a sample size of 2 HIV-1 infected subjects were adequate to determine treatment efficacy with a significance level with alpha = 0.05 and power of test = 0.8. Written informed consent was received from 4 male HIV-1 subjects (Table 1). Inclusion criteria for treatment were: (i) active α1PI below 11 μM; (ii) 1 year history with CD4^+^ lymphocytes at levels ranging between 150 and 300 cells/μl; (iii) absence of symptoms suggestive of HIV-1 disease progression; (iv) adequate suppression of virus (<50 HIV RNA/ml); and (v) history of compliance with antiretroviral medication. Due to the small size of the pilot study and to avoid other complications of pregnancy, only male HIV-1 infected subjects were enrolled. CSL Behring contributed a sufficient quantity of Zemaira® (lot# C405702) for administration of 8 weekly infusions at a dose of 120 mg/kg. Zemaira (α1PI that is purified from plasma) has a half-life after infusion of 4.5 days reaching steady state after 3–4 weeks therapy (Bayer, 2003).

**Table 1:** Enrolled population characteristics at baseline.

**Clinical trial**	**Subject category**	**Age range**	**Active α1PI (μM)**	**CD4 (cells/μl)**	**HIV RNA (copies/ml)**	**Treatment Arm**
**NCT01370018**						
	Pizz-1	50–55	5	743	NA	Prolastin (60 mg/kg)
	Pizz-2	50–55	4	899	NA	Prolastin (60 mg/kg)
	HIV subject-1	46–50	9	297	<400	Zemaira (120 mg/kg)
	HIV subject-2	50–55	7	276	<400	Zemaira (120 mg/kg)
	HIV subject-3	66–70	4	148	<400	Zemaira (120 mg/kg)
	HIV subject-4	50–55	14	445	205	Zemaira (120 mg/kg)
**NCT01731691**						
	uninfected-1	20–25	28	426	NA	Untreated
	uninfected-2	40–45	31	583	NA	Untreated
	uninfected-3	26–30	9	604	NA	Untreated
	uninfected-4	36–40	15	989	NA	Untreated
	HIV subject-1	56–60	11	280	<40	Prolastin-C (120 mg/kg)
	HIV subject-2	46–50	13	318	<40	Prolastin-C (120 mg/kg)
	HIV subject-3	56–60	18	497	<40	Prolastin-C (120 mg/kg)
	HIV subject-4	50–55	9	274	<40	Placebo
	HIV subject-5	46–50	12	501	<40	Placebo
	HIV subject-6	40–45	4	382	<40	Placebo
	HIV subject-7	50–55	16	522	<40	Placebo
	HIV subject-8	56–60	18	510	<40	Placebo

For comparison, we included 2 HIV-1 uninfected patients, both female, with a diagnosis of COPD in the context of genetic α1PI deficiency (PIzz) who were initiating clinical treatment using Prolastin at a dose of 60 mg/kg. Both subjects provided written informed consent.

Each subject was infused at the same time of day and same day of week throughout the study. Blood was collected weekly pre-infusion to monitor changes.

One HIV-1 infected subject who was PPD positive (tuberculosis skin test) had become PPD negative 2 years prior to Zemaira treatment which is clinically interpreted as a loss of immune function. One HIV-1 infected subject reported to the first infusion stating that due to unforeseen circumstances, his antiretroviral medication was interrupted for 4 days. Although there was no fever present or other indication of infection at the time of the first infusion, in follow-up analyses, this subject was found to have pre-treatment serum IL-2 levels of 51 pg/ml (normal is undetectable) and other atypical baseline measures indicative of an inflammatory response which exceeded study inclusion criteria including 454 CD4^+^ cells/μl, 205 HIV RNA copies/ml, and 14 μM α1PI. Thus, blood from this subject was analyzed for the purpose of assessing treatment response in the presence of systemic inflammation.

To determine immunocompetence, CD4^+^ T cells isolated from blood samples acquired pre- and post-Zemaira treatment were analyzed for NFκB activation, cytokine release, and lymphocyte phenotype.

Blood collected at each session was sent to a contractor medical laboratory which provided independent measurement of the complete blood cell count (CBC) with differential, lipid panel, blood chemistry, lymphocyte panel, and HIV RNA. Periodically, kidney (BUN and creatinine) and liver function tests (ALT, AST) were monitored for potential immune complex disease, and all measurements were found to be within the normal range. The study protocol was approved by the Institutional Review Board of Cabrini Medical Center, New York, NY. No adverse effects were reported by any volunteers, and all volunteers remained in the study for the full period with the exception of one HIV-1 subject who agreed to stay in the study for an extra 4 weeks and one PIzz clinic patient who withdrew after the fifth week of the study due to disease-related weakness.

### Clinical Trial NCT01731691

This clinical trial was a double-blind, randomized study. Written informed consent was received from 12 subjects, 8 HIV-1 infected subjects and 4 uninfected controls (Table 1). Inclusion criteria for HIV-1 infected subjects were the same as NCT01370018 with the exception that viral load <1,000 HIV RNA/ml was allowable. Due to a lack of successful accrual, inclusion criteria were modified to allow individuals with 1 year history of CD4^+^ lymphocytes at levels ranging between 200 and 600 cells/μl. Randomization (roll of dice) resulted in 5 HIV-1 infected subjects assigned to placebo treatment and 3 HIV-1 infected subjects assigned to α1PI treatment. HIV uninfected, untreated controls were monitored weekly, and inclusion criteria were the same as for HIV-1 infected subjects excluding HIV-related criteria. All female subjects were required to use contraceptives and were monitored weekly for HCG levels to detect potential pregnancy. Grifols Biotherapeutics contributed a sufficient quantity of Prolastin-C (lot# 26NLK52) for administration of 8 weekly infusions at a dose of 120 mg/kg. Prolastin-C (α1PI that is purified from plasma) is equivalent in activity to Zemaira.

The study protocol was the same as for NCT01370018 and was approved by Copernicus Group Independent Institutional Review Board, Durham, NC. Drug delivery and blood collection were performed at ACRIA, New York, NY. No adverse effects were reported by any volunteers, and all volunteers remained in the study for the full period.

### Serum α1PI Levels

Active and inactive α1PI levels were determined in once-thawed serum samples as previously described (Bristow et al., 1998). Active α1PI represents that fraction of total α1PI that binds to elastase and the residual represents inactive α1PI. Total α1PI levels, measured by ELISA, were confirmed by GeneAidyx, Alachua, FL.

### Lymphocyte Extended Phenotype Analysis

Surface staining on whole blood was performed as previously described (Bristow et al., 2010). Fluorescently conjugated ASR monoclonal antibodies and isotype controls (BD Biosciences) were used to quantitate CD3, CD4, CD8, CD45RA, CD45RO, CXCR4, CCR5, CD34, CD25. Cells were subsequently stained to detect HLE-CS by incubating whole blood for an additional 15 min at 23°C with rabbit anti-HLE (Biodesign, Kennebunkport, ME) or negative control rabbit IgG (Chemicon, Temecula, CA) each of which had been conjugated to Alexa Fluor 647 (Molecular Probes). At least 10,000 cells from each sample were acquired. Markers on cells in the lymphocyte gate were quantitated. In clinical trial NCT01370018, flow cytometry was performed by the investigators. In clinical trial NCT01731691, flow cytometry was performed by a contractor medical laboratory.

### CD4^+^ Lymphocyte Functional Analysis

CD4^+^ lymphocytes were negatively selected from peripheral blood mononuclear cells (PBMC) using magnetic cell sorting as recommended by the manufacturer (Miltenyi Biotec, Auburn, CA). Isolated cells (1 × 10^6^ cells/ml) were cultured in medium containing 10% FBS in 24-well tissue culture plates for 3 days at 37°C, 5% CO_2_, in the presence or absence of stimulation antibodies reactive with CD2, CD3, and CD28 as recommended by the manufacturer (Miltenyi Biotec). Culture supernatants were measured by ELISA as recommended by the manufacturer (R&D Systems, Minneapolis, MN) for IL-2, IL-4, IL-10, and IFNγ.

Harvested CD4^+^ lymphocytes were examined for NFκB phospho-epitope staining by flow cytometry as previously described using phosphoprotein-specific antibodies (BD Pharmingen and BD PhosFlow, San Diego, CA) directly conjugated with Alexa Fluor 647 (Molecular Probes Invitrogen, Carlsbad, CA) (Krutzik and Nolan, 2006).

### sj/β-TREC Ratio Quantification

Thymic function was indirectly calculated in peripheral PBMC DNA using the sj/β-TREC ratio previously described with minor modifications (Ferrando-Martínez et al., 2010). Briefly, the six DβJβ-TRECs from cluster one were amplified together in the same PCR tube, whereas the sj TREC was amplified in a different PCR tube. Twenty-one amplification rounds were performed to guarantee an accurate quantification at the real-time PCR step. All amplicons (DβJβ- and sj-TRECs) were then amplified together in a second PCR using a LightCycler 480 system (Roche). Six μL of a 1/10 dilution of the first-round PCR were amplified in a 20 μL final volume. Forster resonance energy transfer (FRET) specific probes were used for specific detection.

### Statistical Analysis

Correlation and % change from baseline statistical analyses were performed using all available data points in all treatment arms including untreated controls. Most parameters were not normally distributed. Correlation coefficients were determined by Spearman Rank Order unless stated otherwise. Comparison of median values were performed using Mann-Whitney Rank Sum Test.

## Results

### Influence of α1PI Therapy on CD4^+^ T Cell Numbers

**Figure 2:**
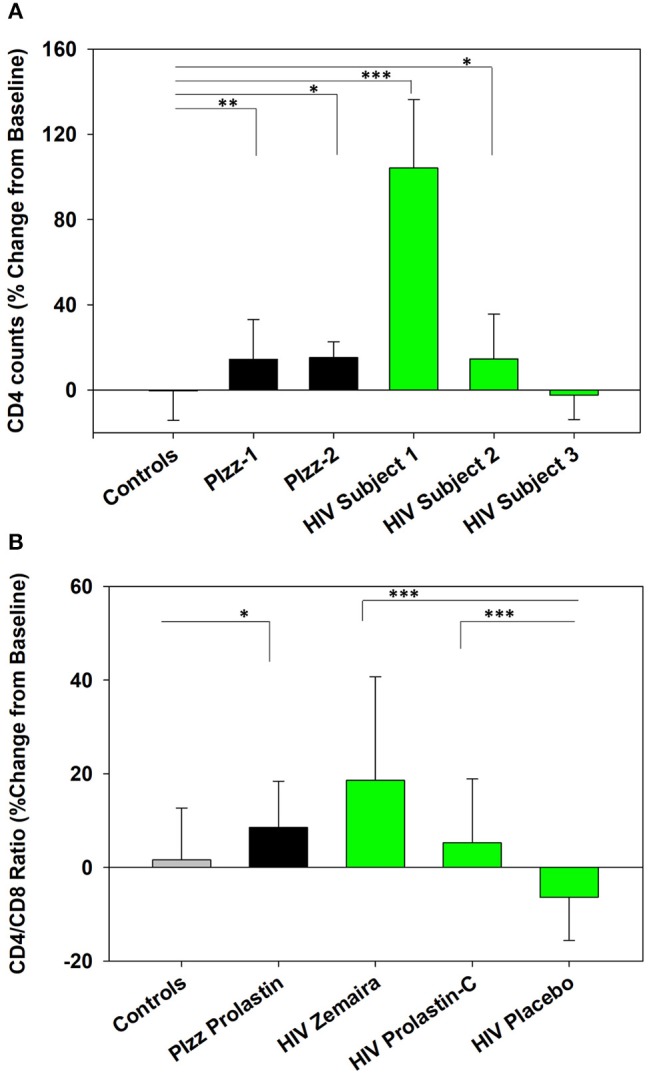
Increased CD4^+^ T cells in α1PI-treated subjects. **(A)** Two Prolastin-treated patients genetically deficient for α1PI (PIzz, black bars) exhibited significantly elevated CD4^+^ T cells (*P* < 0.01 and *P* < 0.04) as compared to four untreated controls (gray bar). Zemaira-treated HIV subject-1 (*P* < 0.001) and HIV subject-2 (*P* < 0.05) (green bars) exhibited significantly elevated CD4^+^ T cells as compared to the four uninfected, untreated controls. HIV subject-3 had lost T lymphocyte-mediated immune response and showed no change in CD4^+^ T cells following Zemaira treatment. **(B)** Two Prolastin-treated PIzz patients exhibited significantly elevated CD4/CD8 ratio (*P* < 0.04, black bars) as compared to four uninfected, untreated controls (gray bar). HIV infected subjects (green bars) exhibited CD4/CD8 ratios that were significantly elevated following treatment with Zemaira (*P* < 0.001, excluding subject-3) and with Prolastin-C (*P* = 0.002) as compared to five subjects treated with placebo. Mean % change from baseline and standard deviations are depicted where % change = 100 × [(Treatment week-Baseline)/Baseline]. Askerisks designate statistically signifant difference (*P < 0.05, **P < 0.01, ***P < 0.001). Data represent nine measurements per subject and were not normally distributed. Comparisons were performed using Mann-Whitney Rank Sum test.

The number of CD4^+^ T cells and CD4/CD8 ratio were significantly increased following clinical initiation of weekly Prolastin therapy in patients with genetic α1PI deficiency (PIzz, *n* = 2, *P* = 0.01, and *P* < 0.04) (Figures 2A,B) (Bristow et al., 2010). Subjects infected with HIV-1 were enrolled in clinical trials to examine the capacity of weekly α1PI to elevate CD4^+^ T cells (Bristow et al., 2010). Following 2 weeks of weekly Zemaira therapy, below normal CD4 counts significantly increased to normal levels of immunocompetent CD4^+^ T cells in 2 subjects (*P* < 0.001 and *P* < 0.05) with no adverse effects (Figure 2A). One HIV-1 subject (HIV subject-3) who had lost the capacity to respond to antigenic challenge (positive PPD followed by negative PPD) showed no increase in CD4^+^ T cells. CD4/CD8 ratio % change from baseline was significantly elevated following Zemaira treatment as well as following Prolastin-C treatment as compared to placebo (Figure 2B).

### Influence of α1PI Therapy on Thymopoiesis

To investigate whether α1PI therapy influences the generation of new CD4^+^ T cells in the thymus, markers of thymopoiesis were measured weekly using peripheral blood from uninfected, untreated subjects and from Prolastin-C-treated and placebo-treated HIV-1 infected subjects. Markers included CD34^+^ cells (pre-thymic progenitor cells), sj/β-TRECs (quantitation of DN to DP maturation), and DPs (pre-SP cells).

The % change from baseline in CD4 counts was not significantly improved in Prolastin-C-treated subjects (Table 2, columns 2, 3, row 2), but increased CD4 counts had been observed with Zemaira and Prolastin treatment (Table 2, columns 4, 5, row 2). In Prolastin-C treatment, CD4% significantly improved relative to placebo treatment (*P* < 0.01, Table 2, columns 2, 3, row 1) as was also observed in Zemaira treatment (Table 2, column 4, row 1. In addition, CD8 counts (*P* < 0.05, Table 2, columns 2, 3, row 4) and CD8% (*P* < 0.001, Table 2, columns 2, 3, row 3) were significantly decreased in Prolastin-C treated subjects as compared to placebo-treated subjects thereby resulting in CD4/CD8 ratios that were significantly higher in Prolastin-C-treated subjects than in placebo-treated subjects (*P* = 0.002, Table 2, columns 2, 3, row 5, Figure 2B) as was also observed with Zemaira and Prolastin treatment (Table 2, columns 4, 5, row 5). CD34^+^ progenitor cells were increased in placebo-treated subjects and to a lesser extent in Prolastin-C treatment (*P* < 0.07, Table 2, columns 2, 3, row 9) although the difference did not reach significance. The relative decrease in % change from baseline in CD34^+^ progenitor cells in Prolastin-C-treated subjects vs. placebo-treated subjects is important to note because the % change from baseline in PIzz Prolastin-treated clinic patients dramatically increased (Table 2, column 5, row 9).

**Table 2:** Effect of α1PI therapy on hematopoietic progenitor cells and T cell phenotypes.

**T cell phenotype**	**NCT01731691**		**NCT01370018**	
	**% change Placebo (HIV)**	**% change Prolastin-C (HIV)**	**% change Zemaira (HIV)**	**% change Prolastin (PIzz)**
CD4%	−5.24 ± 5.10 (38)	−0.49 ± 9.00 (23)^**^	12.81 ± 17.42 (20)	1.85 ± 4.90 (17)
CD4 Abs	−5.19 ± 13.16 (38)	−6.46 ± 16.46 (23)	65.78 ± 53.00 (21)	14.65 ± 16.10 (15)
CD8%	0.92 ± 4.50 (38)	−3.60 ± 3.66 (23)^***^	−2.86 ± 4.57 (20)	−5.97 ± 4.72 (17)
CD8 Abs	1.87 ± 16.97 (38)	−8.14 ± 19.49 (23)*	38.91 ± 33.32 (20)	5.67 ± 11.77 (15)
CD4/CD8 Ratio	−6.33 ± 6.53 (38)	2.92 ± 9.80 (23)^***^	18.31 ± 21.85 (21)	8.70 ± 10.51 (15)
CD3%	−1.86 ± 3.46 (38)	−2.88 ± 2.86 (23)	2.24 ± 3.18 (20)	1.68 ± 2.81 (17)
CD3 Abs	−1.58 ± 15.29 (38)	−8.24 ± 18.73 (23)	48.59 ± 40.16 (20)	14.44 ± 14.72 (15)
CD4^+^CD8^+^ events	2.15 ± 39.41 (36)	28.05 ± 135.53 (23)	N.D.	−14.62 ± 73.26 (10)
%CD34^+^	158.34 ± 263.23 (36)	20.13 ± 103.46 (14)	N.D.	2,187.14 ± 2,770.97 (10)
CD3^+^HLE^+^ events	22.61 ± 92.70 (30)	257.63 ± 705.84 (16)	N.D.	N.D.
sj/β-TRECs	3,115.11 ± 9810.35 (30)	3,456.23 ± 7,463.45 (19)	N.D.	N.D.
Lymphocytes	−2.77 ± 20.4 (30)	18.18 ± 32.92 (23)^**^	46.60 ± 44.08 (27)	6.75 ± 10.5 (10)

Values depicted represent means and standard deviations of % change from baseline and are color coded with green representing positive changes and red representing negative changes. The number of observations in placebo-treated (n = 5 subjects), Prolastin-C-treated (n = 3 subjects), Zemaira-treated (n = 3 subjects), and Prolastin-treated subjects (n = 2 subjects) is represented in parenthesis (n). Measurements were not performed in some cases (N.D.). Parameters were not normaly distributed and medians were compared using Mann-Whitney Rank Sum Test. Askerisks designate a statistically significant difference between placebo and Prolastin-C (*P < 0.05, ^**^P < 0.02, ^***^P < 0.001). Zemaira-treated subjects and PIzz clinic patients are presented, but were not compared due to the differences in disease parameterss, treatment dose, and lack of HIV-1 infected untreated controls.

In addition to change from baseline, relationships between markers of thymopoiesis and cell phenotypes were altered by Prolastin-C treatment as follows:

### CD4/CD8 Ratio (Table 3 Part 1, Column 1)

In uninfected controls, as expected, increased CD4/CD8 ratio correlated with increased absolute CD4^+^ T cells and with increased DPs. In HIV-1 infected subjects, Prolastin-C treatment, but not placebo treatment, corrected the relationship between the CD4/CD8 ratio and absolute CD4^+^ T cells as well as the relationship between CD4/CD8 ratio and DPs to resemble that of uninfected controls (Table 3 Part 1, column 1, rows 2 and 8).

**Table 3:** Effect of α1PI therapy on the relationships between progenitor cells and T cell phenotypes.

		**1**			**2**			**3**		
		**N-CD4/CD8 Ratio**	**P-CD4/CD8 Ratio**	**Pro-CD4/CD8 Ratio**	**N-%CD34^**+**^**	**P-%CD34^**+**^**	**Pro-%CD34^**+**^**	**N-CD4^**+**^CD8^**+**^ DP events**	**P-CD4^**+**^CD8^**+**^ DP events**	**Pro-CD4^**+**^CD8^**+**^ DP events**
**PART 1**										
1	CD4%	0.847	0.791	0.935	0.375			0.621		0.67
		0.000027	2.00E-07	2.00E-07	0.0246			0.0000552		0.000219
		36	42	25	36			36		25
2	CD4 Abs	0.724		0.563	0.472	−0.534	−0.401	0.534		0.85
		2.00E-07		0.00352	0.00381	0.00036	0.0468	0.000869		2.00E-07
		36		25	36	41	25	36		25
3	CD8%	−0.961	−0.72	−0.78	−0.36			−0.898		−0.762
		2.00E-07	2.00E-07	2.00E-07	0.0313			2.00E-07		2.00E-07
		36	72	25	36			36		25
4	CD8 Abs		−0.567	−0.796	0.35	−0.379				−0.439
			0.000102	2.00E-07	0.0366	0.0149				0.0281
			42	25	36	41				25
5	CD4/CD8 Ratio							0.872		0.726
								2.00E-07		7.59E-06
								36		24
6	CD3%			−0.424			0.498		0.433	−0.722
				0.0347			0.0116		0.00549	6.66E-06
				25			25		40	25
7	CD3 Abs	0.559	−0.444	−0.67	0.506	−0.441		0.415		
		0.000435	0.00341	0.000219	0.00175	0.00411		0.0122		
		36	42	25	36	41		36		
8	CD4^+^CD8^+^ DP events	0.872		0.726		0.66	−0.481			
		2.00E-07		7.59E-06		2.30E-06	0.0152			
		36		24		40	25			
9									0.602	
	%CD34%								4.27E-05	
									40	
10	CD3^+^HLE^+^ Events	0.424					0.437	0.492	0.424	
		0.0113					0.0365	0.00285	0.00826	
		35					23	35	38	
		**4**			**5**			**6**		
		**N-TRECs**	**P-TRECs**	**Pro-TRECs**	**N-Active a1PI**	**P-Active a1PI**	**Pro-Active** a1PI	**N-Inactive a1PI**	**P-Inactive a1PI**	**Pro-Inactive a1PI**
**PART 2**										
1	CD4%						0.738		−0.669	−0.668
							2.00E-07		3.05E-07	0.000167
							26		43	26
2	CD4 Abs		0.0206			0.669	0.606		−0.449	−0.579
			0.00658			4.64E-07	0.00107		0.00265	0.00195
			33			42	26		43	26
3	CD8%					0.644	−0.841			0.589
						3.41E-06	2.00E-07			0.00162
						42	26			26
4	CD8 Abs		0.227		−0.422	0.671	−0.657	0.385		0.442
			0.000263		0.0107	4.01E-07	0.000252	0.0204		0.0237
			33		36	42	26	36		26
5	CD4/CD8 Ratio					−0.347	0.863		0.408	−0.723
						0.0263	2.00E-07		0.00831	4.29E-06
						41	25		41	25
6	CD3%				−0.455	0.563	−0.682	0.332	−0.513	0.404
					0.00548	0.000116	0.0000956	0.0479	0.000489	0.0407
					36	42	26	36	43	26
7	CD3 Abs		0.21			0.672	−0.537			
			6.26E-04			3.11E-07	0.00484			
			33			42	26			
8	CD4^+^CD8^+^ DP events				0.326		0.768			−0.607
					0.0526		2.00E-07			0.00134
					36		25			25
9										0.554
	%CD34%									0.00514
										24
10	CD3^+^HLE^+^ events				0.449		−0.476	−0.355		
					0.00709		0.0163	0.0365		
					35		25	35		

*Subject designations (top row) represent uninfected, untreated (“N” violet, n = 4 subjects), placebo-treated (“P” light orange, n = 5 subjects), and Prolastin-C-treated (“Pro” blue, n = 3 subjects). Boxes containing correlations are color-coded with red representing negative correlations, green representing positive correlations, white representing no correlation, and gray representing irrelevant correlations. Light green represent correlations that approached, but did not reach significance. The values depicted within each box represent correlation coefficients (r, top row), significance (P, middle row), and number of observations (n, bottom row)*.

In uninfected controls, CD4/CD8 ratio was not correlated with absolute CD8^+^ T cells, but in placebo treatment and Prolastin-C treatment increased CD4/CD8 ratio was correlated with decreased absolute CD8^+^ T cells (Table 3 Part 1, column 1, row 4). Further, in uninfected controls, increased CD4/CD8 ratio was correlated with increased absolute CD3^+^ T cells, but in placebo treatment and Prolastin-C treatment, CD4/CD8 ratio was correlated with decreased absolute CD3^+^ T cells (Table 3 Part 1, column 1, rows 6 and 7). These results suggest that Prolastin-C treatment caused an increase in CD4/CD8 ratio by increasing DPs and CD4^+^ T cells, not by decreasing absolute CD3^+^ or CD8^+^ T cells.

### CD34^+^ Progenitor Cells (Table 3 Part 1, Column 2)

In uninfected controls, increased CD34^+^ progenitor cells (% lymphocyte gate) were correlated with increased absolute CD4^+^ T cells, increased absolute CD3^+^ T cells, and increased absolute CD8^+^ T cells (Table 3 Part 1, column 2, rows 2, 4, 7). In contrast, in placebo treatment, increased CD34% was correlated with decreased absolute CD4^+^ T cells, decreased absolute CD3^+^ T cells, and decreased absolute CD8^+^ T cells (Table 3 Part 1, column 2, rows 2, 4, 7).

In Prolastin-C-treated subjects, there was no correlation between CD34% and absolute CD3^+^ T cells or absolute CD8^+^ T cells, but increased CD34% was correlated with decreased absolute CD4^+^ T cells (Table 3 Part 1, column 2, rows 2, 4, 7). Further, in uninfected controls, CD34% was not correlated with DPs. In placebo treatment, increased CD34% was correlated with increased DPs, whereas in Prolastin-C treatment, increased CD34% was correlated with decreased DPs (Table 3 Part 1, column 2, row 8). While Prolastin-C treatment did not significantly increase or decrease CD34^+^ progenitor cells (Table 2, columns 2, 3, row 9), these correlation comparisons suggest that Prolastin-C treatment altered the relationship between CD34^+^ progenitor cells and DPs, CD3^+^, and CD8^+^ T cells, but not between CD34^+^ progenitor cells and CD4^+^ T cells.

### DPs (Table 3 Part 1, Column 3)

In uninfected controls, increased DPs were correlated with increased CD4% and absolute CD4^+^ T cells. In placebo treatment there was no correlation, but in Prolastin-C treatment, the relationship between the DPs and CD4% and absolute CD4 counts was corrected to resemble that of uninfected controls (Table 3 Part 1, column 3, rows 1 and 2).

In uninfected controls, increased DPs were correlated with decreased CD8%. In HIV-1 infected subjects, Prolastin-C treatment, but not placebo treatment, corrected the relationship between the DPs and CD8% to resemble that of uninfected controls. Prolastin-C treatment also caused decreased DPs to become correlated with decreased absolute CD8 counts (Table 3 Part 1, column 3, rows 3 and 4).

In uninfected controls, there was no correlation between DPs and CD3%. However, in placebo treatment, increased DPs were correlated with increased CD3%. In contrast, in Prolastin-C treatment, increased DPs were correlated with decreased CD3% (Table 3 Part 1, column 3, row 6). In uninfected controls, increased DPs were correlated with increased absolute CD3^+^ T cells, but in HIV-1 infected subjects, this correlation was absent (Table 3 Part 1, column 3, row 7). CD3% is the percent of CD3^+^ T cells in the lymphocyte gate. If DPs are not correlated with CD3% (where the number of total lymphocytes is the denominator), but are positively correlated with CD3^+^ T cells, then DPs are not related to the total number of lymphocytes. In placebo treatment, where DPs were positively correlated with CD3%, but not with CD3^+^ T cells, DPs were negatively related to the number of lymphocytes, i.e., the more DPs, the fewer lymphocytes. In Prolastin-C treatment, where DPs were negatively correlated with CD3%, but not correlated with CD3^+^ T cells, DPs were positively related to the number of lymphocytes, i.e., the more DPs, the more lymphocytes. This interpretation is supported by the increase in lymphocytes in Prolastin-C treatment, Zemaira treatment, and Prolastin treatment as compared with placebo treatment (Table 2, row 12).

In uninfected controls, increased DPs were correlated with increased CD4/CD8 ratio. In HIV-1 infected subjects, Prolastin-C treatment, but not placebo treatment, corrected the relationship between the DPs and CD4/CD8 ratio to resemble that of uninfected controls (Table 3 Part 1, column 3, row 5).

In uninfected controls and Prolastin-C treatment, there was no correlation between DPs and CD34^+^ progenitor cells. In contrast, in placebo treatment, increased DPs were correlated with increased CD34^+^ progenitor cells (Table 3 Part 1, column 3, rows 9).

These combined results suggest that Prolastin-C treatment altered the relationship between DPs and CD34^+^ progenitor cells, between DPs and CD4/CD8 ratio, between DPs and absolute CD4^+^ T cells, between DPs and absolute CD8^+^ T cells, but not between DPs and absolute CD3^+^ T cells suggesting Prolastin-C treatment fostered the development of DPs to become CD4^+^ T cells.

### sj/β-TRECs (Table 3 Part 2, Column 4)

In uninfected controls and in Prolastin-C treatment, sj/β-TRECs were not correlated with absolute CD3^+^, CD4^+^, or CD8^+^ T cells. Yet, in placebo treatment, increased sj/β-TRECs were correlated with increased absolute CD3^+^, CD4^+^, and CD8^+^ T cells (Table 3 Part 2, column 4, rows 2, 4, 7). These results suggest that Prolastin-C treatment corrected the relationship between sj/β-TRECs and absolute CD3^+^, CD4^+^, or CD8^+^ T cells to resemble that of uninfected controls.

### Active and Inactive α1PI (Table 3, Part 2, Columns 5 and 6)

In uninfected controls, neither active nor inactive α1PI were correlated with absolute CD4^+^ T cells. In placebo and Prolastin-C treatment, increased active α1PI was correlated with increased absolute CD4^+^ T cells and inactive α1PI was correlated with decreased absolute CD4^+^ T cells (Table 3, Part 2, columns 5 and 6, rows 1 and 2).

In uninfected controls, increased active α1PI was correlated with decreased absolute CD8^+^ T cells and decreased CD3% whereas increased inactive α1PI was correlated with increased CD8^+^ T cells and increased CD3% (Table 3, Part 2, columns 5 and 6, rows 4 and 6). In uninfected controls, there was no correlation between active and inactive α1PI and CD4/CD8 ratio, yet in placebo treatment, increased active α1PI was correlated with decreased CD4/CD8 ratio and in Prolastin-C treatment, the opposite relationship was found with increased active α1PI being correlated with increased CD4/CD8 ratio (Table 3, Part 2, columns 5 and 6, row 5).

With regard to progenitor cells, in uninfected controls, increased active α1PI was correlated with increased DPs, but there was no relationship between DPs and inactive α1PI. In placebo treatment, there was no relationship between DPs and active or inactive α1PI. However, in Prolastin-C treatment, increased active α1PI was correlated with increased DPs, and inactive α1PI was correlated with decreased DPs (Table 3, Part 2, columns 5 and 6, row 8).

In all 3 arms of the study, there was no relationship between active α1PI and CD34^+^ progenitor cells, but this was not so for inactive α1PI. In uninfected controls and placebo treatment, there was no relationship between inactive α1PI and CD34^+^ progenitor cells, but in Prolastin-C treatment, increased inactive α1PI was correlated with increased CD34^+^ progenitor cells (Table 3, Part 2, columns 5 and 6, row 9). These combined results support the interpretation that active α1PI actively increases CD4/CD8 ratio without increasing DPs, increasing CD34^+^ progenitor cells, or increasing sj/β-TRECS.

### CD3^+^HLE^+^ T Cells (Table 3, Parts 1 and 2, Row 10)

CD3^+^HLE^+^ T cells are a principal target for active α1PI. In uninfected controls, increased CD3^+^HLE^+^ T cells were correlated with increased CD4/CD8 ratio, but this relationship was not observed in HIV-1 infected subjects with placebo or Prolastin-C treatment.

In uninfected controls and placebo treatment, CD3^+^HLE^+^ T cells were not correlated with CD34^+^ progenitor cells, yet in Prolastin-C treatment increased CD3^+^HLE^+^ T cells were correlated with increased CD34^+^ progenitor cells (Table 3, Part 1, columns 1 and 2, row 10).

In uninfected controls and in placebo treatment, increased CD3^+^HLE^+^ T cells were correlated with increased DPs, but in Prolastin-C treatment there was no relationship between CD3^+^HLE^+^ T cells and DPs (Table 3, Part 1, column 3, row 10).

There was no relationship between CD3^+^HLE^+^ T cells and sj/β TRECs in any arm (Table 3, Part 2, column 4, row 10).

In uninfected controls, increased CD3^+^HLE^+^ T cells were correlated with increased active α1PI and decreased inactive α1PI. In placebo treatment, there was no relationship between CD3^+^HLE^+^ T cells and active or inactive α1PI. However, in Prolastin-C treatment, increased CD3^+^HLE^+^ T cells were correlated with decreased active α1PI (Table 3, Part 2, columns 5 and 6, row 10).

These results support the interpretation that CD3^+^HLE^+^ T cells interact with active α1PI to regulate DPs and CD4/CD8 ratio supporting our previous findings that α1PI is rate limiting in HIV-1 disease for generation of CD4^+^ T cells and that replacing α1PI allows HIV-1 infected patients to resemble uninfected controls (Bristow et al., 2012).

## Discussion

Although the number of HIV-1 subjects receiving α1PI therapy in these two clinical trials is small, the evidence suggests that α1PI participates in regulating the maturation of DPs to SP CD4^+^ T cells thereby elevating CD4/CD8 ratio.

Data from Table 2 show that Prolastin-C treatment significantly increased CD4/CD8 ratio and total lymphocytes, and improved CD4%, while significantly decreasing CD8% and CD8 counts. Data from Table 3 show that Prolastin-C treatment corrected correlations from abnormal to normal between CD4/CD8 ratio and CD4 counts as well as between CD4/CD8 ratio and DPs. These results suggest that Prolastin-C treatment caused an increase in CD4/CD8 ratio by increasing absolute CD4^+^ T cells and by decreasing absolute CD8^+^ T cells.

Data from Tables 2, 3 show that Prolastin-C treatment altered relationships between CD34% and DPs, CD34% and CD3%, CD34% and absolute CD3^+^ T cells, CD34% and absolute CD8^+^ T cells, but not between CD34% and CD4% or absolute CD4^+^ T cells. These results suggest that Prolastin-C treatment caused an increase in CD4/CD8 ratio and improved CD4% without increasing CD34%, but by modifying the relationship between CD34% as well as DPs with absolute CD8^+^ T cells.

Data from Table 3 show that Prolastin-C treatment corrected to normal relationships between DPs and CD4/CD8 ratio, between DPs and CD4%, between DPs and absolute CD4^+^ T cells, between DPs and CD8%, between DPs and CD34%, but not between DPs and CD3% or absolute CD3^+^ T cells. These results suggest that Prolastin-C treatment caused an increase in CD4/CD8 ratio and improved CD4% without increasing DPs, but by modifying the relationship between DPs and absolute CD4^+^ T cells and absolute CD8^+^ T cells.

Data from Table 3 show that Prolastin-C treatment corrected to normal relationships between TRECs and absolute CD3^+^ T cells, TRECs and absolute CD4^+^ T cells, and TRECs and absolute CD8^+^ T cells. These results suggest that Prolastin-C treatment caused an increase in CD4/CD8 ratio and improved CD4% without increasing TRECs, but by modifying the relationship between TRECs and absolute CD4^+^ T cells, CD8^+^ T cells and CD3^+^ T cells.

Data from Table 3 show that Prolastin-C corrected to normal relationships between active α1PI and absolute CD8^+^ T cells, between active α1PI and CD3%, between active α1PI and DPs, but not between active α1PI and absolute CD4^+^ T cells or CD34%. These results suggest that Prolastin-C treatment caused an increase in CD4/CD8 ratio and improved CD4% at a step between CD34% and CD4^+^ T cells.

Considering that during maturation, CD3 is expressed simultaneously with the TcR, the lack of a significant sj/β-TREC change from baseline in Prolastin-C-treated subjects is reasonable since there was also not a significant absolute CD3^+^ T cell change from baseline. In placebo-treated HIV-1 infected subjects, increased sj/β-TRECs were correlated with increased absolute CD3^+^, CD4^+^, and CD8^+^ T cells, but this correlation was not observed for uninfected, untreated controls. Prolastin-C therapy corrected the realtionship between sj/β-TRECs and absolute CD3^+^, CD4^+^, and CD8^+^ T cells to correspond to uninfected controls (no correlation) supporting evidence that α1PI, without significantly increasing sj/β-TRECs, DPs, CD34%, or absolute CD3^+^, CD4^+^, or CD8^+^ T cells is integral to the maturation of CD4^+^ T cells, clearly from DPs (Bristow et al., 2012).

The difference between Zemaira and Prolastin-C treatment to elevate CD4 counts vs. decrease CD8 counts was attributed to the difference in subject populations since both drug lots were found to have the same activity during FDA approval. The Zemaira trial enrolled homosexual, HIV-1 infected individuals that were compared to uninfected, α1PI genetically deficient individuals with no evidence of systemic inflammation, whereas the Prolastin-C trial enrolled, almost entirely, IV drug users, all of whom exhibited consistent or sporadically elevated CRP, a marker of inflammation. Inflammation is known to elevate cytokines and enzymes that could influence thymopoiesis, and evidence has established that inflammation is inversely correlated with DPs (Bristow et al., 1998; Ferrando-Martinez et al., 2009). That α1PI therapy increased CD4/CD8 ratio by either increasing the number of absolute CD4^+^ T cells or decreasing the number of absolute CD8^+^ T cells, depending on patient population, suggests, unsurprisingly, that there are additional factors that determine the phenotype of SP cells.

Evidence published in the early 1980's demonstrated that four T cell alloantigens in mice encoded by genes on chromosome 12 are expressed in discrete T cell phenotypes, suggesting these genes participate in thymopoiesis (Owen and Riblet, 1984). The four mouse genes located near the immunoglobulin heavy chain gene are distinctly expressed on what were termed, at that time, Tind (T helper, CD4^+^, OKT4^+^, Lyt-1^+^), Tsu (T suppressor, CD8^+^, Lyt-2^+^3^+^), Tthy (thymocytes, DP, Lyt-1^+^2^+^3^+^), and Tpre (Tthy precursors) (Owen et al., 1982; Owen, 1983). It was widely and enthusiastically anticipated that like immunoglobulin gene rearrangement, the four T cell alloantigen genes rearranged with the immunoglobulin heavy chain gene to form a T cell antigen receptor, the existence and structure of which was hotly disputed to be an immunoglobulin-like protein named IgT (Marchalonis, 1975; Janeway et al., 1976; Marchalonis and Cone, 2002). However, within a year, the mouse T cell antigen receptor genes were cloned, sequenced, and found to be encoded on chromosomes 6, 13, and 14, not chromosome 12 (Hedrick et al., 1984; Yanagi et al., 1984; Brenner et al., 1986). This surprising revelation shattered the hypothesis that the four mouse T cell alloantigens were part of the T cell antigen receptor and regrettably provided no information as to what is encoded on chromosome 12 that discriminates T cell functional phenotypes. Ultimately, it was shown that the four monoclonal antibodies that discriminated the four mouse T cell alloantigens all bind specifically to human α1PI, a protein encoded on mouse chromosome 12 and on human chromosome 14 near the immunoglobulin heavy chain gene (Dembic et al., 1985; Bristow and Flood, 1993). Whereas, there is one human gene that expresses α1PI, there is a cluster of 6 expressed mouse α1PI genes (UnitproKB–P07758). These combined data are a compelling indication that mouse α1PI isoforms and human α1PI regulate thymopoiesis. Further, by inactivating human α1PI, 3F5 HIV-1 antibody has been shown to inhibit the generation of new CD4^+^ T cells.

As a natural host for HIV-1, chimpanzees (*Pan troglodytes*) can be infected with HIV-1, but they neither develop AIDS, nor permanently lose their CD4^+^ T cells. A primary reason for the relative resistance of these primates to develop AIDS is that monoclonal antibody 3F5 does not bind to chimpanzee α1PI due to a single amino acid difference caused by one nucleotide difference in human α1PI that lies within the 3F5-binding region of the protein (Bristow et al., 2012).

A strategy is proposed in which HIV-1 vaccination includes induction of an antibody that blocks 3F5 activity, thereby preventing the autoimmune influence that produces acquired α1PI deficiency and, as a consequence, inhibits CD4^+^ T cell maturation. Such a vaccine would attempt to prevent AIDS in addition to current attempts to prevent HIV-1 infection, a situation which might allow humans to simulate the illness experienced by chimpanzees infected with HIV-1 and to survive infection relatively unscathed.

Treating HIV-1 patients for the purpose of elevating CD4^+^ T cells using mutated α1PI might be feasible, potentially using the chimpanzee sequence, yet there is a risk that mutated α1PI might also stimulate antibodies against the mutated sequence. A less risky treatment is to develop small molecules that target HLE-CS and produce the same cellular response as α1PI.

Our group has identified a panel of orally-available small molecules that bind to HLE-CS and induce cellular locomotion. In several pre-clinical mouse studies, our lead molecule has been demonstrated to increase the number of CD4^+^ T cells with no untoward side effects in two mice strains with varying disease states (unpublished results pending completion of pre-clinical studies). The development of small molecules in these studies are encouraging that, for the first time, treatment of secondary immunodeficiency may be possible.

## Footnote

Some data from an earlier clinical trial presented herein were previously published in an open-access eBook chapter, “α_1_Antitrypsin therapy increases CD4^+^ lymphocytes to normal values in HIV-1 patients.” In: M. Alfano, editors. Soluble factors mediating innate immune responses to HIV infection, Bentham Science Publishers, and are exhibited here in an alternative format for comparison to data from a second, more extensively investigated, previously unpublished clinical trial.

## Data Availability Statement

The datasets generated for this study are available on request to the corresponding author.

## Ethics Statement

The studies involving human participants were reviewed and approved by IRB, Cabrini Medical Center, New York, NY; Copernicus Group Independent Institutional Review Board, Durham, NC. The patients/participants provided their written informed consent to participate in this study.

## Author Contributions

CB and RW designed the studies. CB performed the correlation analysis, interpreted data, and wrote the manuscript. SF-M, ER-M, and ML provided the TRECs measurements and analysis.

### Conflict of Interest

SF-M was employed at the Vaccine Research Center, NIH, during the time the research was performed and subsequently took a position at Medimmune. The remaining authors declare that the research was conducted in the absence of any commercial or financial relationships that could be construed as a potential conflict of interest.

## Abbreviations

α1PI: α1proteinase inhibitor, α1antitrypsin, Alpha-1
bNAbs: broadly neutralizing antibodies
HLE-CS: human leukocyte elastase on the cell surface
LDL-RFM: LDL receptor family members
TRECs: T cell receptor excision circles
DN: double negative CD4^−^CD8^−^ T cells
DP: double positive CD4^+^CD8^+^ T cells
SP: single positive CD4^+^ or CD8^+^ T cells.
